# Experimental Study on the Roundness of Deep Holes in 7075 Aluminum Alloy Parts by Two-Dimensional Ultrasonic Elliptical Vibration Boring

**DOI:** 10.3390/mi14122185

**Published:** 2023-11-30

**Authors:** Shuaikun Yang, Jinglin Tong, Ziqiang Liu, Yanqiu Ye, Haojie Zhai, Hongqing Tao

**Affiliations:** School of Mechanical and Power Engineering, Henan Polytechnic University, Jiaozuo 454003, China; 212105020019@home.hpu.edu.cn (S.Y.); a2016476371a@163.com (Z.L.); 212105020061@home.hpu.edu.cn (Y.Y.); 212205020047@home.hpu.edu.cn (H.Z.); thq2244813051@home.hpu.edu.cn (H.T.)

**Keywords:** ultrasonic elliptical bore boring, 7075 aluminum alloy, deep hole precision machining, surface roundness, deep hole pipes

## Abstract

The 7075 aluminum alloy deep hole pipe finds extensive applications in the aerospace industry due to its remarkable attributes, such as high strength, exceptional wear resistance, and favorable mechanical properties. However, traditional boring processes for 7075 aluminum alloy deep hole pipes tend to generate elevated cutting forces, potentially leading to deformation issues in these deep holes. In response to these challenges, this study introduces a novel approach involving the use of a two-dimensional ultrasonic elliptical vibration tool. This tool features a single excitation asymmetric structure and aims to enhance the deep hole machining process in 7075 aluminum alloy. The research methodology involved several key steps. First, theoretical analysis and simulation were performed to study the motion trajectory of the cutting edge of the tool. Second, practical experiments were conducted comparing two-dimensional ultrasonic elliptical vibration boring with conventional boring for 7075 aluminum alloy deep hole pipes. The results demonstrate that, in contrast to conventional boring, two-dimensional ultrasonic vibration boring could achieve a maximum reduction of 54.1% and an average reduction of 50.4% in the roundness value of the deep holes. The impact of machining parameters on deep hole roundness is assessed through experimental analysis, leading to the determination of optimal processing parameters. In summary, this experimental research has a certain reference significance for the application of 7075 aluminum alloy deep hole parts in the aerospace field.

## 1. Introduction

With the development of ultrasonic machining technology and the progress of the aerospace industry, the machining accuracy requirements for the main structure and body parts of aircraft engines are constantly improving. The optimization and upgrading of machining technology and equipment is an inevitable trend. At present, the processing technology and equipment for aluminum alloy deep holes have received widespread attention in the aerospace manufacturing industry [1,2,3], mainly reflected in the important value of aluminum alloy deep holes in specific applications of aerospace equipment, and their processing accuracy directly affects their working performance and the overall safety of aerospace equipment. Therefore, with the improvement of performance requirements for aviation equipment, the technical requirements for these parts are also [4,5].

The 7075 aluminum alloy cylinders have been widely used in the modern aerospace industry and arms industry because of their compact structure, light weight, good comprehensive performance, and material savings. However, due to their high plasticity and easy deformation, it is difficult for the surface quality and accuracy of the workpieces to meet the requirements in ordinary turning, which restricts the development of aviation- and aerospace-related technical equipment. The aerospace industry has strict requirements for the selection of process parameters, resulting in low production efficiency and yield of 7075 aluminum alloy cylinders [6,7,8]. Therefore, the improvement and upgrading of 7075 aluminum alloy deep hole processing technology and equipment are of great significance for improving its performance and production efficiency.

Ultrasonic vibration cutting is a processing method that uses the piezoelectricity of piezoelectric ceramics to convert electrical signals into super-high-frequency mechanical vibration signals, which are transmitted to tools or workpieces for processing. It was first proposed and verified by Japanese scholar Junichiro Kumabe [9] and has received attention from scholars from various countries. Afterward, a large number of domestic and foreign scholars conducted theoretical analyses and experimental research on ultrasonic machining technology, further improving the progress of ultrasonic machining technology [10,11,12], and ultrasonic vibration machining technology has gradually become a new precision machining technology.

Many scholars in the manufacturing field have conducted theoretical analyses and experimental research on ultrasonic vibration machining. Yingshuai Xu et al. [13] used ultrasonic-vibration-assisted cutting technology to study the turning of 304 austenitic stainless-steel workpieces. The results show that with the help of ultrasonic vibration and appropriate ultrasonic amplitude in turning, the cutting force was significantly reduced. At the same time, cutting parameters have a significant impact on cutting force. If the correct cutting parameters are selected, UAT will achieve more ideal machining results. Neeraj Deswal et al. [14] conducted ultrasonic-vibration-assisted turning research on magnesium AZ31B alloy and compared it with CT in terms of processing force, processing temperature, tool wear, chip morphology, surface roughness, microstructure, and microhardness. The results indicate that compared with CT, the processing force and surface roughness of UVAT are significantly reduced. The processing temperature obtained during UVAT was higher than that of CT, and side wear was observed during CT. The chip thickness of UVAT was higher than that of CT process. The grain size of UVAT is finer than that of coarse grain, and its microhardness is higher, while the microhardness of CT is lower. Compared with CT process, processing magnesium AZ31B alloy in the UVAT process can improve processing performance. Bo Li et al. [15] used laser ultrasonic-vibration-assisted turning technology to study the turning of SiCp/AI composite materials, established an L-UVAM finite element model, and conducted experiments. The experimental results show that compared with traditional machining, L-UVAM had better surface quality and fewer broken particles and defects on the machined surface. The simulation results are in good agreement with the cutting experimental results. The research of the above researchers indicates that ultrasonic vibration machining has become a precision machining technology, and researchers have demonstrated that this technology is superior to ordinary machining in terms of cutting force, machining temperature, tool wear, chip morphology, surface roughness, microstructure, and microhardness.

In aerospace industry applications, Veiga F. [16] proposed a predictive force model based on commercial control support without external vibration device chip breaking function, and the average error of the model was verified by experiments to be only 10%. The functionality of this model is very attractive for commercial applications. The model promotes chip breakage through low-frequency vibration boring and can also be applied with different materials and parameters. In addition, considering the vibration amplitude of half of the feed per tooth, the roughness can be increased by 33%. The work of Zou et al. [17] performed low-frequency vibration-assisted drilling (LFVAD) operations on newly developed CFRP/Al cocuring materials. The mechanism and feasibility of using LFVAD to control machining defects are discussed theoretically and experimentally, and the effect of vibration amplitude (VA) on cutting response is emphatically studied. The results show that, compared with conventional drilling, LFVAD can reduce the average thrust and drilling temperature, but the maximum thrust increases with an increase in VA, and the maximum thrust is reached when VA is ~30 μm. At the same time, because of its superior chip breaking effect, LFVAD can reduce the scratches on the surface of the hole wall, so as to improve the drilling accuracy. On the other hand, due to the reduction in cutting temperature, the damage area of the lower interface can be reduced. The results show that the LFVAD method has higher hole accuracy and lower interface damage response for CFRP/Al cocuring materials, and it has greater advantages.

In the field of cutting aluminum alloys, researchers have also conducted different aspects of processing research. Lacalle et al. [18] studied conventional drilling and peck drilling and concluded that this is the most widely used solution for aluminum alloy drilling at present, and they pointed out its existing problems. They proposed a new alternative, low-frequency-assisted drilling (LFAD), which performs frequency vibrations (50 to 100 Hz). The process of drilling chip formation assisted by FC/Al laminates with low-frequency vibration is modeled analytically and compared with conventional aluminum drilling process. The results show that chip segmentation reduces the temperature rise and avoids the geometrical quality problems and burr formation in the final hole during drilling. Heng Luo et al. [19] investigated the influence of ultrasonic vibration parameters on the machining performance of 7050-T651 aluminum alloy. Their research encompassed both simulation and experimental analyses, revealing that ultrasonic vibration turning offers substantial reductions in cutting force and temperature when compared with conventional turning methods. Jinglin Tong’s [20] work delved into the impact of ultrasonic elliptical vibration turning on the surface microstructure of aluminum alloys. The findings underscored the notable influence of ultrasonic amplitude on the surface microstructure of these alloys. Hélder Puga et al. [21] studied the effect of aluminum alloy ultrasonic-assisted turning material processing on improving roughness values. The study showed that the intermittent effect of ultrasonic vibration on the tool can improve surface quality, reduce surface roughness, and reduce the maximum peak height by 76%. They also found that the cutting shape has changed.

Xianfu Liu [22,23] conducted a research study focused on the microtexturing of one-dimensional ultrasonic vibration machining of end faces. Their experimental work confirmed the feasibility of microtexture machining on end faces. Following this, they undertook a simulation-based investigation to model the controllable generation of textures on cylindrical surfaces. The findings of this study revealed that various machining parameters, including clearance angle, spindle speed, and vibration amplitude, significantly influence the resulting texture patterns. Through careful adjustment of clearance angles, spindle speeds, and vibration amplitudes, it is possible to achieve different intersecting states. This, in turn, leads to changes in the size, shape, and distribution of microdepressions. Notably, when the head radius and feed rate are increased, the width of the depressions and the spacing between adjacent depressions along the feed direction also exhibit corresponding increases.

Single-dimensional ultrasonic vibration machining technology has certain limitations and cannot meet the continuously improving machining quality requirements. Therefore, scholars have conducted extensive research on multidimensional ultrasonic vibration machining technology. The implementation of two-dimensional ultrasonic vibration is mostly achieved by synthesizing multiple one-dimensional vibrations in different directions [24]. The amplitude and phase difference between ultrasonic excitation sources [10] can affect the elliptical motion trajectory of the cutting edge. Ming Zhou et al. [25] introduced an innovative ultrasonic elliptical vibration cutting apparatus that integrates bending and longitudinal vibrations. They assessed the device’s performance through experimental research, revealing substantial reductions in tool wear when compared with conventional cutting techniques. Jinchuan Yang et al. [26] proposed a method for optimizing ultrasonic elliptical vibration, which solved the difficulties of coupling ultrasonic elliptical vibration and the low controllability of elliptical trajectories. Through experiments, it was proven that the tool designed using this optimization method achieved good elliptical trajectory control and improved cutting performance of the tool. Yin et al. [27] developed and optimized an efficient single-excitation ultrasonic elliptical vibration device featuring an asymmetric structure. Experimental studies have shown that this device can enhance the surface quality of high tungsten alloy materials and reduce tool wear.

The study of ultrasonic vibration turning cannot be separated from the study of surface quality, including surface roughness and many other aspects. Nestler Andreas et al. [28] employed ultrasonic vibration applied to the cutting tool along the feed, radial, and cutting directions. They conducted experiments involving ultrasonic vibration turning on particle-reinforced aluminum matrix composites, focusing on analyzing alterations in surface morphology and roughness of the machined surfaces. Jay Airao et al. [29] introduced an innovative research approach that integrates ultrasonic vibration with microlubrication (MQL) and CO_2_ to enhance the machinability of Ti-6Al-V. Experimental investigations have indicated that this approach leads to a substantial reduction in specific cutting energy and enhances the sustainability of Ti-6Al-V machining. Teimouri et al. [30] conducted experimental research on 7075 aluminum alloy using ultrasonic vibration with a rotating tool during turning. Their data analysis revealed that this approach can effectively reduce cutting forces and improve surface roughness.

In addition to being applied to outer circular surfaces, two-dimensional ultrasonic elliptical vibration has also been extensively studied in large aperture ratio inner hole parts. Dong et al. [31] developed an ultrasonic elliptical vibration boring apparatus and carried out experimental research on deep hole parts made from high-strength steel 18Cr2Ni4WA. The experimental findings highlighted that increasing the excitation voltage effectively diminishes cutting forces, improves surface roughness, and enhances the stability of the boring process. In comparison with traditional boring methods, ultrasonic elliptical vibration boring demonstrates significant reductions in boring forces, surface roughness, chip length, and boring bar vibrations. Sui et al. [32,33] explored the application of the axial ultrasonic-vibration-assisted boring (AUVB) method for machining deep holes with a very large aspect ratio (greater than 4) in Ti20Al6V aviation materials. Their findings indicate that the AUVB method effectively reduces aperture errors by 50%, limits vibration amplitudes to 20–25%, and lowers the overall surface roughness of the workpiece to below 0.8 μm. Additionally, it facilitates the transition of the surface residual stress state from tensile to compressive at specific speeds. Lu et al. [34] introduced an ultrasonic vibration-assisted boring approach, demonstrating its effectiveness in reducing boring forces and surface roughness, particularly when utilizing longitudinal torsional vibration. In comparison with conventional boring methods, this technique resulted in a notable reduction in boring forces ranging from 38.04% to 43.77% and a decrease in surface roughness between 25.48% and 41.47%. Ngo et al. [35] also proposed a vibration device design method that can be applied to the vibration assisted boring (UAB) process. Research has shown that compared with CB, the surface roughness value of machined holes using UAB is reduced by up to 95%, with a confidence level of 50%.

The research conducted above has highlighted the superiority of ultrasonic-assisted machining compared with traditional machining methods. Researchers have substantiated this through theoretical models, simulations, and experimental studies. However, there are still challenges in effectively addressing the difficulties associated with hole machining in demanding aviation applications due to the nature of hard-to-cut materials and stringent machining standards. The existing research on ultrasonic boring only proposes a device for ultrasonic assisted boring holes, and it has been proven that this method can improve surface roughness, but further research has not been conducted in other areas.

Hence, this article seeks to devise a two-dimensional, ultrasonic elliptical-assisted boring apparatus to effectively tackle the challenges posed by demanding aviation applications, characterized by hard-to-cut materials and stringent machining standards, and is committed to solving the classic challenge in the traditional boring field of how to ensure machining accuracy and reduce roundness error.

## 2. Analysis of Kinematics Characteristics of Deep Hole Boring

The mechanical cutting edge movement trajectory mainly includes spiral movement: in deep hole boring, the cutting edge is usually fed with spiral movement. This mode of motion helps to improve cutting stability and processing efficiency. The spiral motion trajectory of the cutting edge is usually parallel to the axis of the workpiece, but there can also be a certain spiral angle to better control the cutting process. Radial motion: In addition to spiral motion, the cutting edge also has radial motion. We attach a 20,000 Hz ultrasonic vibration to the tool handle, which can be used to adjust the contact position of the cutting edge with the workpiece surface for more precise machining. The radial motion also helps to control the clearance between the cutting edge and the hole wall, avoiding friction between the cutting tool and the hole wall.

During ultrasonic vibration machining, the workpiece or tool will be subjected to high-frequency ultrasonic vibration for cutting. During two-dimensional ultrasonic elliptical vibration turning, the cutting tool undergoes periodic high-frequency vibration in the X, Y, and Z directions. This results in a more intricate tool tip trajectory compared with conventional turning, as it experiences the influence of two excitation sources. Furthermore, the dynamic characteristics of the tool tip during turning exhibit variations depending on the specific cutting and acoustic parameters employed. These substantial alterations in the tool tip’s motion trajectory in two-dimensional ultrasonic elliptical vibration turning, as opposed to conventional turning, have a direct impact on the material removal rate of the workpiece, consequently leading to transform in the roundness dimensions achieved after deep hole machining.

### 2.1. Design and Analysis Testing of Two-Dimensional Ultrasonic Vibration Boring Tool

The amplitude converter is an important component of the ultrasonic vibration turning system. When the amplitude converter is an asymmetric structure, some composite vibrations may occur, such as longitudinal torsional composite, longitudinal bending composite, and bending torsional composite. By utilizing the asymmetry of the horn structure and adjusting the structural parameters, an ultrasonic elliptical vibration form can be formed at the turning tool tip.

**Figure 1:**
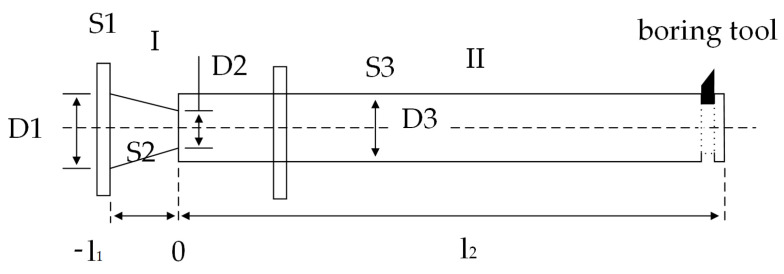
Schematic diagram of longitudinal bending composite vibration amplitude converter.

As shown in Figure 1, the first section of the horn is a conical horn with a relatively large shape factor. In order to improve the stability of the horn, a cylindrical horn is used in the second section. It is assumed that the composite horn is composed of uniform materials, without considering the mechanical loss and damping vibration; the ultrasonic wave propagates along the axis in the horn, and the horn meets the Wave equation:

(1) $$\frac{\partial^{2}\xi }{\partial x^{2}}+\frac{1}{S}\cdot \frac{\partial S}{\partial x}\cdot \frac{\partial \xi }{\partial x}+k^{2}\xi =0$$

In the formula, ξ=ξ(x) is the particle displacement function, k=ω/c, ω is the circular frequency, and c is the propagation speed of longitudinal waves in a thin rod, $c=\sqrt{E/\rho }$; S=S(x) is the cross-sectional area.

For the first section of the horn, the change in the conical section is

(2) $$S\left(x\right)=S_{2}{\left(1+\alpha x\right)}^{2}$$

In the formula, *S*_2_ is the cross-sectional area of the small end of the conical segment, $\alpha =\frac{N-l}{Nl}$, and *N* is the area coefficient, $N=\frac{D_{2}}{D_{1}}$. Substituting Equation (1) yields

(3) $$\frac{\partial^{2}\xi }{\partial x^{2}}+\frac{2\alpha }{1+\alpha x}\cdot \frac{\partial \xi }{\partial x}+k^{2}\xi =0$$

For the second section of the amplitude converter, $S\left(x\right)=S_{3}$, $\frac{\partial S}{\partial x}=0$, substituting into Equation (1) can obtain

(4) $$\frac{\partial^{2}\xi }{\partial x^{2}}+k^{2}\xi =0$$

The general solution of the equation is $\xi \left(x\right)=A\cos\left(kx+kl_{2}\right)+B\sin\left(kx+kl_{2}\right)$.

According to the boundary conditions $\xi \left(x\right)|{}_{x=-l_{2}}=\xi_{1},\frac{\partial \xi \left(x\right)}{\partial x}|{}_{x=-l_{2}}=0$, $A=\xi_{1},B=0$ can be obtained.

The displacement distribution function can be obtained from known conditions and boundary conditions as

(5) $$\xi \left(x\right)=\frac{\xi_{1}\cos\left(kl_{2}\right)}{1+\alpha x}\left[\cos\left(kx\right)-\frac{\alpha }{k}\sin\left(kx\right)\right]$$

According to the displacement boundary conditions, the amplification coefficient of the amplitude transformer can be obtained, $M_{P}=\left|{\left(\frac{D_{3}}{D_{1}}\right)}^{2}\cdot \frac{D_{1}}{D_{2}}\sin kl_{1}\right|$.

Given the design frequency f = 20 kHz and sound speed = 5200 m/s of the ultrasonic turning tool, the material selection is 45 # steel. From the formula derived above, it can be seen that *l*_1_ = 74 mm, *l*_2_ = 480 mm, *D*_1_ = 40 mm, *D*_2_ = 20 mm, *D*_3_ = 30 mm. The turning tool is connected to the cylindrical horn with a fixing nut.

The material properties of 45 steel: density σ = 7.9 × 10^3^ (kg/m^3^), Young’s modulus E = 20.92 × 10^10^ (N/m^2^), tensile strength σ_b = 61 × 10^7^ (N/m^2^), velocity of longitudinal wave sound in rod c = 5169 (m/s).

The material selection requirements of the horn mainly include (1) the loss of the material in the operating frequency range is small; (2) high material fatigue strength and small acoustic impedance, so as to obtain a large vibration speed and displacement amplitude; (3) easy to machine; (4) when carrying out liquid treatment, it is also required that the material used in the radial surface of the amplitude transformer is corrosion-resistant; and (5) the horn material should be forged, and the fiber elongation direction is consistent with the acoustic transmission line to improve the fatigue resistance and acoustic performance of the horn.

Generally speaking, titanium alloy has the best performance, but it is expensive, and the machining is difficult. Aluminum alloy is cheap and easy to machine, but its resistance to ultrasonic cavitation corrosion is poor. Steel is cheap, easy to process, and the loss is general. Brass wears out a lot. In summary, the material model is 45 steel.

**Figure 2:**
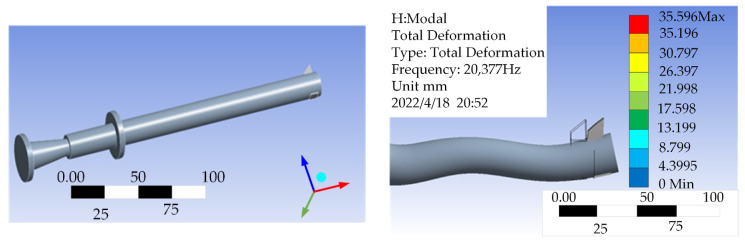
Modal analysis of the two-dimensional ultrasonic amplitude modulation system.

As shown in Figure 2, ANSYS 2020 software was used to simulate the designed turning tool. Firstly, a modal analysis was conducted on the two-dimensional ultrasonic elliptical vibration turning tool. The material of the hornwas selected as 45 # steel, with a dynamic search frequency range of 20–40 kHz. The results are shown in Figure 2. It can be seen that there are longitudinal and bending vibrations at the blade tip, with a frequency of 20,377 Hz, which has an error of 1.89% compared with the design value of 20 kHz. The error is mainly caused by material properties and grid division issues, which meet the design requirements.

**Figure 3:**
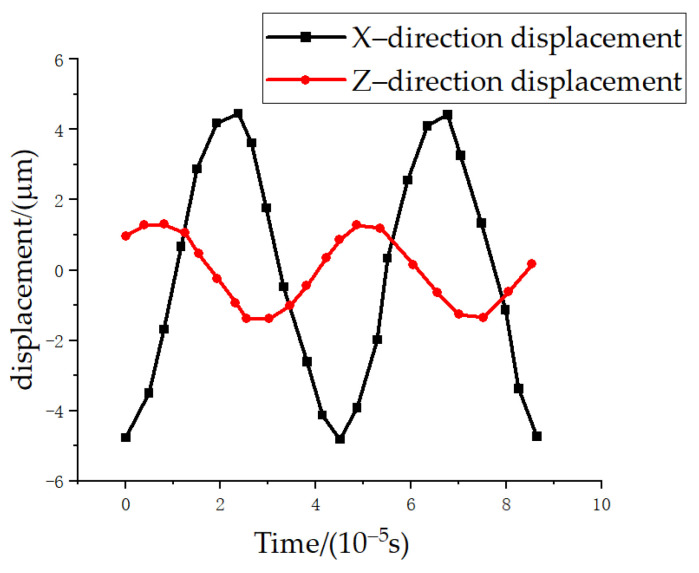
Ultrasonic vibration tool tip displacement trajectory diagram.

In order to analyze the vibration of asymmetric horn under one-dimensional longitudinal excitation, the statics analysis of the horn was carried out using finite element analysis software. Using free grid division, apply 0.001 cos (2 π) to the horn × the trajectory curve of the turning tool under unidirectional excitation of 20,000 t, as shown in Figure 3. From Figure 3, it can be seen that the tool exhibits alternating displacement in both the X and Z directions, with an amplitude ratio of approximately 1.2:4, and the displacement pattern at the tool tip is approximately elliptical.

**Figure 4:**
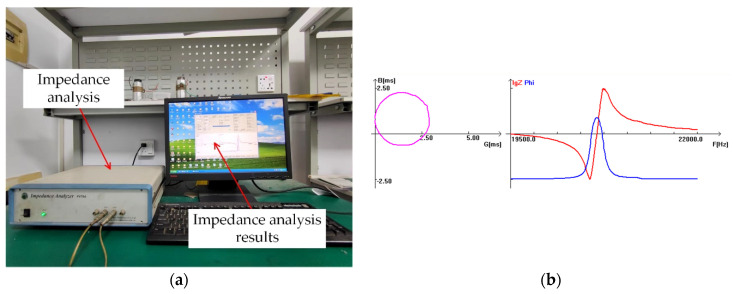
Impedance measurement diagram.

As shown in Figure 4a, the impedance analyzer was used to analyze and test the turning tool. As shown in Figure 4b, the measured resonant frequency of the turning tool is 20.234 kHz, which has a little error with the design value. It is within the acceptable range, indicating that the design is feasible.

**Figure 5:**
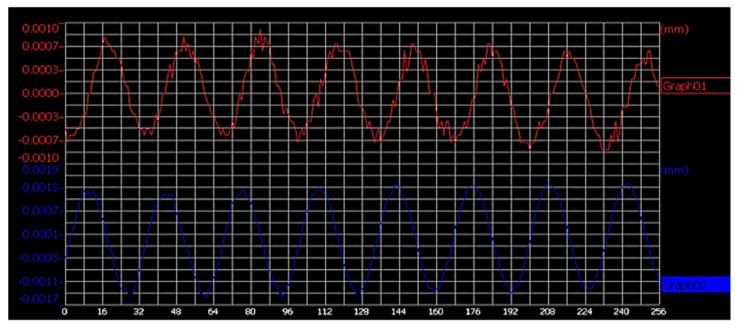
Ultrasonic amplitude test results.

To gauge the amplitude of the custom-designed turning tool, a KEYENCE laser displacement sensor (LK-G10) was employed. Figure 5 illustrates a series of displacement waveforms obtained from the measurements.

### 2.2. Kinematics Model of Ultrasonic Elliptical Vibration Turning

**Figure 6:**
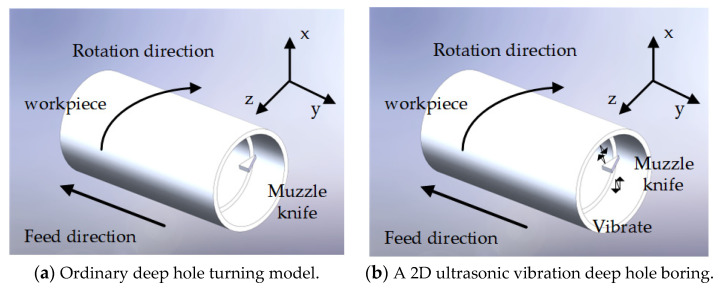
Model of Ultrasonic Vibration Turning of Inner Holes.

In order to study the motion characteristics of two-dimensional ultrasonic vibration, it is first necessary to understand the cutting edge motion characteristics of ordinary boring deep holes. As shown in Figure 6a, in ordinary boring machining, the tool is not subjected to any other active excitation, and Equation (5) is its cutting edge trajectory equation:

(6) $$\left\{ \begin{array}{l} X\left(t\right)=nf_{r}t/60\\ Y\left(t\right)=\left(r+a_{p}\right)\cdot \cos\left(2\pi nt/60\right)\\ Z\left(t\right)=\left(r+a_{p}\right)\cdot \sin\left(2\pi nt/60\right) \end{array}\right.$$

In the formula, the variables are defined as follows: 

*X*: Displacement in the feed direction.

*Y*: Displacement in the direction of cutting depth.

*Z*: Displacement in the direction of cutting speed.

*r*: The radius of the deep hole.

*f_r_*: Feed rate.

*a_p_*: Cutting depth.

*t*: Cutting time.

*n*: Spindle speed.

In two-dimensional ultrasonic elliptical vibration turning, aside from its rotation and relative feeding motion with respect to the workpiece, the tool also undergoes ultrasonic vibrations in both the *X* and *Z* directions. The vibration equation is as follows:

(7) $$\left\{ \begin{array}{l} X\left(t\right)=A_{X}\cdot \sin\left(2\pi ft+\theta \right)\\ Z\left(t\right)=A_{Z}\cdot \sin\left(2\pi ft\right) \end{array}\right.$$

In the formula, $A_{X}$ and $A_{X}$ represent the amplitudes in the *X* and *Y* directions, while θ indicates the phase difference between the *X* and *Y* directions. Since this experiment employs a single-excitation elliptical vibration turning tool, theoretically, θ remains a constant value.

In the simultaneous Equations (6) and (7), the motion equations governing the cutting edge in the two-dimensional elliptical vibration turning of deep holes are as follows:

(8) $$\left\{ \begin{array}{l} X\left(t\right)=\left(r+a_{p}\right)\cdot \cos\left(2\pi nt/60\right)+A_{X}\cdot \sin\left(2\pi ft+\theta \right)\\ Y\left(t\right)=nf_{r}t/60\\ Z\left(t\right)=\left(r+a_{p}\right)\cdot \sin\left(2\pi nt/60\right)+A_{Z}\cdot \cos\left(2\pi ft\right) \end{array}\right.$$

**Figure 7:**
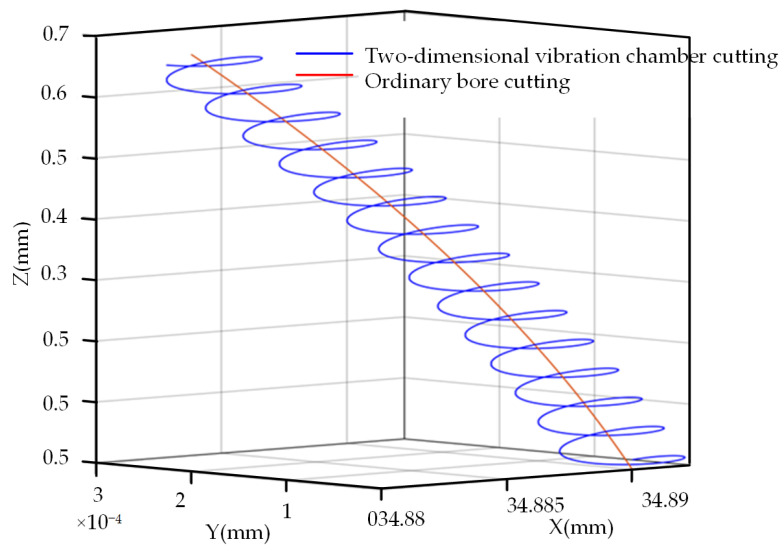
Two-dimensional ultrasonic elliptical vibration cutting and traditional cutting tool tip motion trajectory.

Figure 7 shows the motion trajectory of the cutting edge.

From Figure 7, it is evident that the tool tip’s motion path during two-dimensional ultrasonic elliptic vibration turning exhibits a pronounced elliptical vibration pattern, accompanied by a partial rotation overlap phenomenon in the trajectory. At this juncture, the vibration velocity (*v*) of the cutting edge comprises the feed direction velocity ($v_{f}$), cutting velocity ($v_{F}$), and the vibration velocity components ($v_{x}$ and $v_{z}$) associated with both directions. In this experiment, a single-excitation asymmetric two-dimensional ultrasonic elliptical vibration boring tool is employed, making it possible to disregard the influence of the phase difference on the vibration trajectory of the cutting edge. As a result, variations in spindle speed, feed rate, ultrasonic amplitude, and ultrasonic vibration frequency applied to the cutting depth will collectively affect the vibration trajectory of the cutting edge.

### 2.3. Simulation of Cutting Edge Motion Trajectory

Based on the preceding analysis, it becomes evident that the motion trajectory of the cutting edge in two-dimensional ultrasonic vibration turning for deep hole machining is notably intricate. Ultrasonic amplitude, spindle speed, feed rate, cutting depth, and ultrasonic vibration frequency collectively influence the vibration trajectory of the cutting edge. Furthermore, it is worth noting that the motion trajectory of the cutting edge exerts a direct impact on the surface morphology and quality of the machined deep hole surface. Hence, there is a need to investigate the motion trajectory of cutting edges.

The motion trajectory of the cutting edge was analyzed using a single-factor experimental approach, and the experimental plan is detailed in Table 1.

The equations governing the motion of the cutting edge in two-dimensional ultrasonic elliptical vibration turning are simulated using MATLAB 2020a software, and various motion trajectories of the cutting edge under different parameters are obtained through simulation. The inner diameter of the workpiece being machined is R = 70 mm.

1. The Effect of Ultrasonic Amplitude on Cutting Edge Trajectory

**Figure 8:**
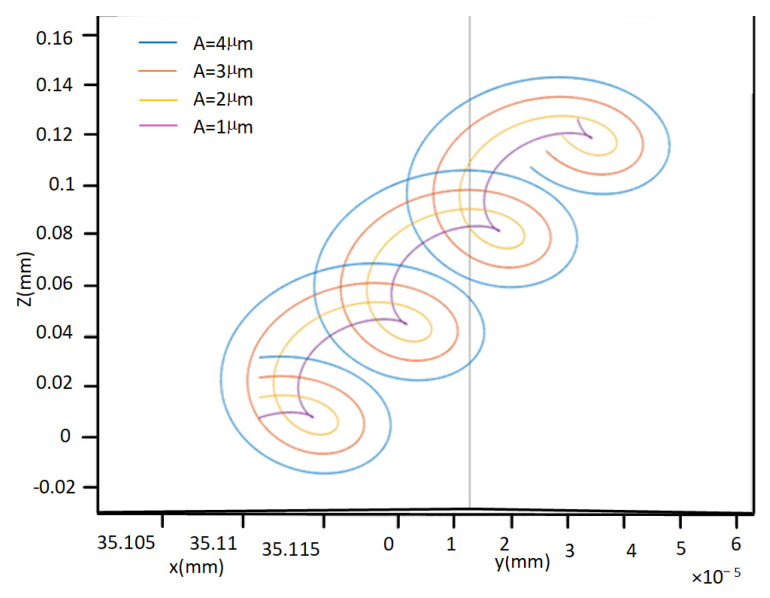
Motion trajectories of different amplitudes.

As shown in Figure 8, the magnitude of the ultrasonic amplitude has a significant impact on the motion trajectory of the cutting edge. When the amplitude A=4μ, the phenomenon of rotary cutting is the most obvious, and even the overlapping of cutting edge trajectories between adjacent vibration periods occurs. When the amplitude A=1μ, the phenomenon of rotary cutting is the least obvious. A smaller ultrasonic amplitude will result in a smaller rotational cutting area generated by the cutting edge.

2. The Influence of Rotational Speed on Cutting Edge Trajectory

**Figure 9:**
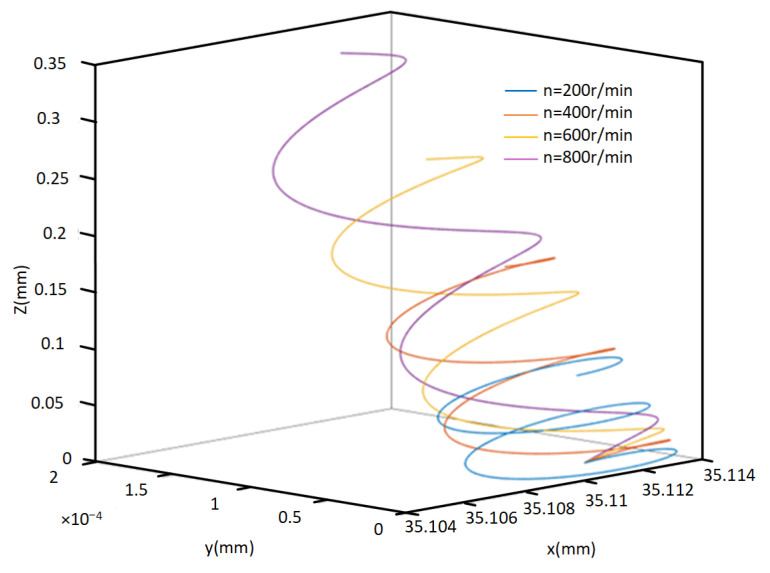
Motion trajectories at different rotational speeds.

As depicted in Figure 9, it is evident that the rotational speed has a relatively significant impact on the motion trajectory of the cutting edge. The figure clearly illustrates that the influence of rotational speed primarily manifests in the direction of cutting velocity. Furthermore, the cutting velocity ($v_{F}$) also increases as the rotational speed rises. Once the cutting speed surpasses the vibration speed ($v_{Z}$) in the *Z* direction, the condition for tool–workpiece separation, SR < 1, ceases to be valid in the *Z* direction, consequently diminishing the effectiveness of two-dimensional ultrasonic vibration turning. Hence, opting for a higher rotational speed that complies with the tool–workpiece separation conditions during actual machining not only enhances machining efficiency but also ensures the efficacy of two-dimensional ultrasonic vibration turning.

3. The Influence of Cutting Depth on Cutting Edge Trajectory

**Figure 10:**
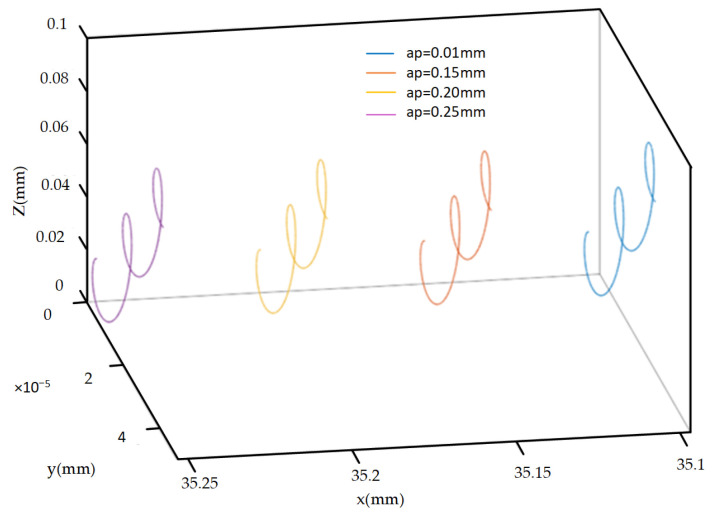
Trajectories of different cutting depths.

As shown in Figure 10, as the cutting depth increases, the motion trajectory of the cutting edge does not show significant changes in shape and features, only the initial position changes.

4. The Influence of Feed Rate on Cutting Edge Trajectory

**Figure 11:**
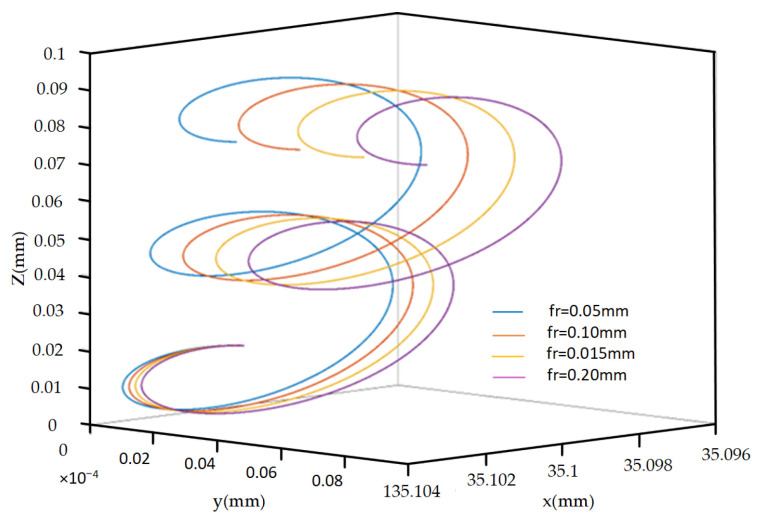
Trajectories of different feed rates.

As shown in Figure 11, with the same other parameters, as the feed rate increases, the displacement of the cutting edge motion trajectory in the *X* direction increases slightly, but there is no significant change in the other directions.

## 3. Design of Experiments and Method

### 3.1. Construction of Experimental Platform

**Figure 12:**
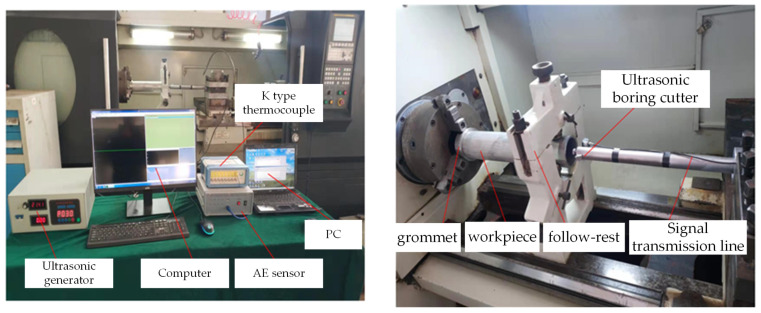
Two-dimensional ultrasonic vibration turning test platform.

Mount and secure the two-dimensional ultrasonic vibration turning tool onto the machine tool’s tool holder. The turning tool is linked to the ultrasonic generator via a cable, and the electrical signal produced by the power supply is transmitted to the transducer within the two-dimensional ultrasonic elliptical vibration turning tool to generate the necessary vibration signal. One end of the workpiece is securely clamped using a three-jaw chuck, while the other end is affixed to a tool holder. The primary equipment and models employed are detailed in Table 2, and the experimental setup is illustrated in Figure 12.

### 3.2. Test Materials

The chamber knife model employed in the experiment is DCGT070204-AK, and its specific parameters are provided in Table 3. The workpiece is a cylindrical tube made of 7075 aluminum alloy, with an outer diameter of R = 80 mm, wall thickness of h0 = 5 mm, length of L0 = 430 mm, and a depth-to-diameter ratio exceeding 5, measuring 6.14. This workpiece qualifies as a deep hole cylinder. Tensile strength: 524 Mpa, yield strength: 455 Mpa, modulus of elasticity: E:71 Gpa, hardness: 150 HB, density: 2.81 g/cm^3^, Poisson’s ratio: 0.33.

**Figure 13:**
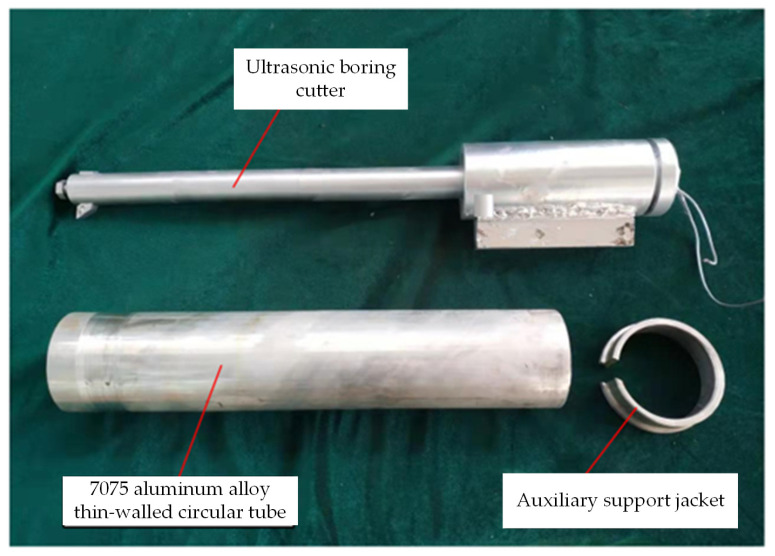
Physical picture of the workpiece and tool.

Figure 13 depicts the physical object of a 7075 aluminum alloy pipe alongside an auxiliary support structure. Given the thin wall thickness of the selected 7075 aluminum alloy tube, which is only 5mm, clamping it directly onto a three-jaw chuck could result in excessive clamping force, leading to workpiece deformation. Conversely, insufficient clamping force may cause instability in the workpiece axis, thereby affecting test results. Therefore, an auxiliary support jacket is utilized at the end of the three-jaw chuck to secure and safeguard the workpiece, preventing direct deformation due to excessive clamping force. Considering the workpiece’s substantial length of 430mm, which can induce significant oscillation during rotation, a tool holder is employed at the turning end to axially stabilize the workpiece.

### 3.3. Design of Experimental Plan

During the process of performing two-dimensional ultrasonic elliptical vibration boring on 7075 aluminum alloy inner holes, analysis reveals that alterations in factors such as feed rate (*f_r_*), spindle speed (*n*), ultrasonic amplitude (*A*), and cutting depth (*a_p_*) can exert a certain influence on the experimental outcomes. To delve into the impact of these various factors on the surface roughness and roundness of deep hole machining, both single-factor experiments and orthogonal experimental schemes were devised. The orthogonal-factor table is presented in Table 4, while detailed parameters for the single-factor experiment are provided in Table 5.

The numerical control lathe produced by Shenyang Machine Tool Co., Ltd. (Shenyang, China) was CAK50186di, and the limit speed was 900 r/min. Considering the parameter limitations of the lathe used in the experiment and the impact of radial runout when the workpiece rotates with the spindle, the aluminum alloy hole wall is first roughened and precision machined before the experimental processing of the workpiece to ensure that there is no situation where the tool cannot cut due to workpiece runout during the experiment.

### 3.4. Test Result Detection

**Figure 14:**
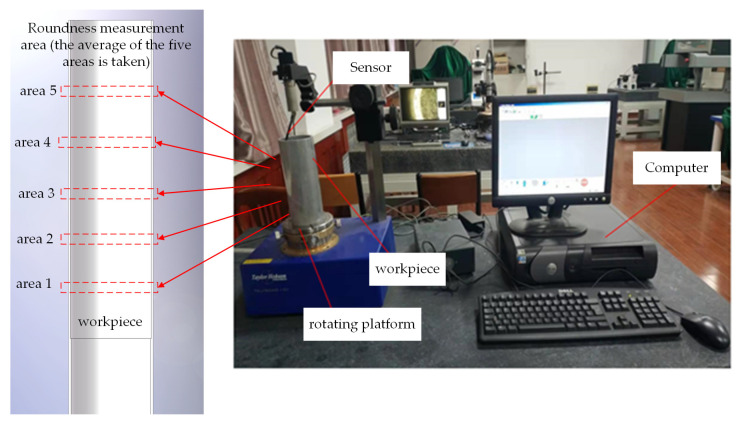
Roundness inspection site map.

Due to the fact that the experimental processing object is aluminum alloy deep holes, the measurement of the inner wall roundness of the workpiece should be measured without damaging the integrity of the workpiece, which is relatively complex compared with ordinary outer surfaces. As shown in Figure 14, a Taylor Hobson roundness meter was used to measure the roundness of the machined surface. The device is a Taylor Hobson Talyrond roundness measuring instrument manufactured by Swedish precision parts manufacturer Taylor Hobson, model number Talyrond 130.

## 4. Experimental Results and Analysis

### 4.1. Analysis of Roundness Single-Factor Test Results

1. The influence of Feed Rate on Roundness

**Figure 15:**
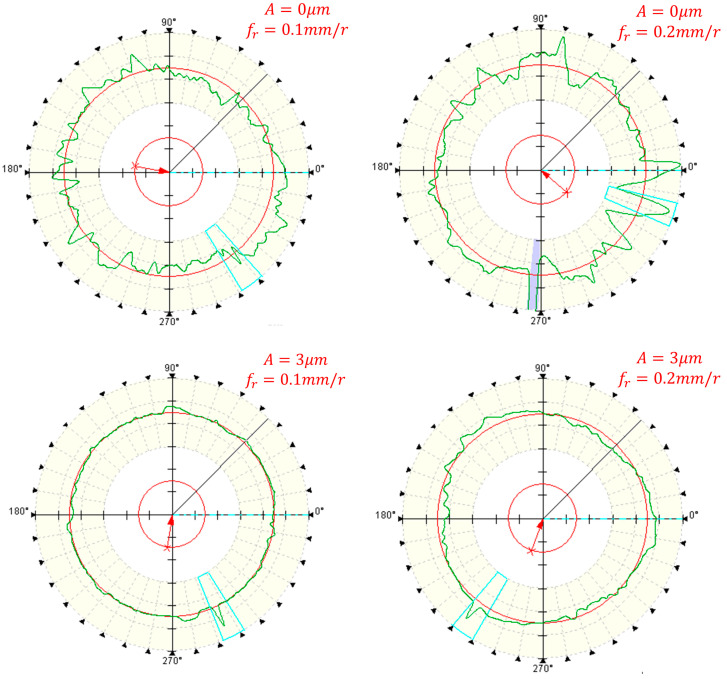
Measurement diagram of deep hole roundness under different feed rates.

**Figure 16:**
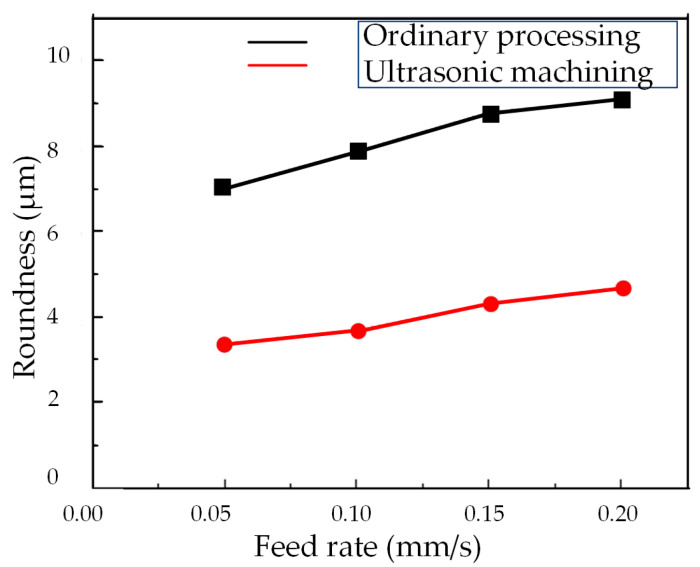
Changing the feed rate.

Figure 15 the roundness error curves of deep hole surfaces under conventional and ultrasonic machining conditions at different feed rates. And Figure 16 shows the influence of feed rates on the roundness of workpiece machined surfaces.

Figure 15 demonstrates that the roundness curve achieved through the two-dimensional ultrasonic vibration turning of deep holes exhibits smoother contours and smaller deviations when contrasted with conventional turning methods. From Figure 16, it becomes evident that under identical parameter conditions, the roundness measurements of deep holes in two-dimensional ultrasonic vibration boring are smaller than those in conventional boring. Two-dimensional ultrasonic vibration turning can achieve a maximum reduction of 53.2% in the roundness of the machined surface, with an average reduction of 51.1%.

According to the previous theoretical analysis, this is because the cutting force generated by ultrasonic vibration has a pulse characteristic, which can reduce the energy intake of the cutting system, thereby suppressing the chatter of the tool bar during deep hole machining, increasing the critical cutting depth and significantly improving the stiffness of the machining system, thereby improving the machining accuracy and reducing the roundness of deep holes. Similar to conventional machining, the roundness of the deep hole surface in two-dimensional ultrasonic vibration turning also exhibits an increase as the feed rate rises, albeit with a marginal change. This phenomenon occurs because as the feed rate increases, the cutting width approaches the critical cutting width, leading to greater tool bar vibration. Consequently, opting for a lower feed rate during machining can enhance the roundness of the machined surface.

2. The Effect of Rotational Speed on Roundness

**Figure 17:**
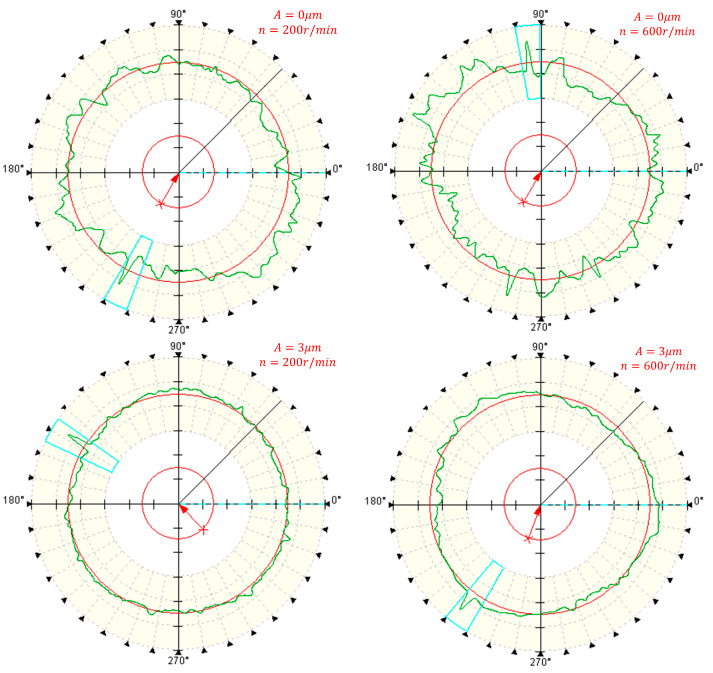
Roundness measurement of deep holes at different rotational speeds.

**Figure 18:**
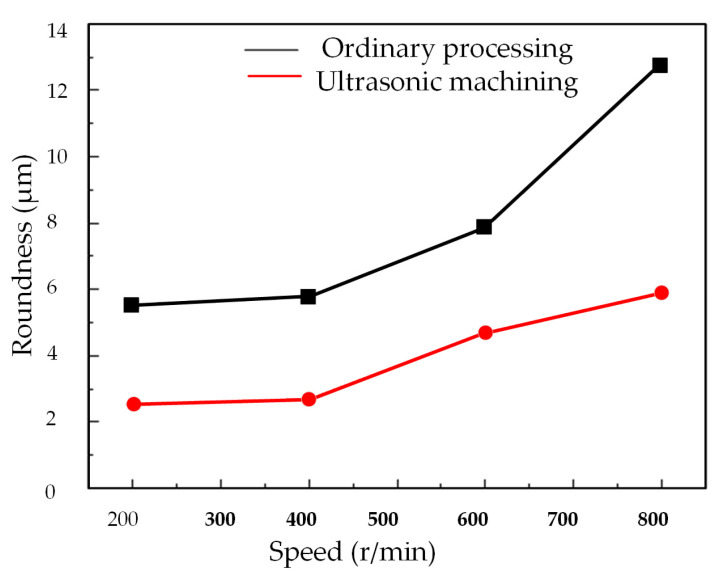
Changing the speed.

Figure 17 and Figure 18 illustrates the impact of spindle speed on the roundness of deep holes. It is noticeable that irrespective of the machining method, when the speed remains below 400 r/min, there is no substantial alteration in the roundness of deep holes with increasing speed. However, once the speed surpasses 400 r/min, the roundness of conventionally machined surfaces notably increases. Similarly, the surface roundness in two-dimensional ultrasonic vibration machining also rises with an increase in rotational speed.

The trajectory analysis of two-dimensional ultrasonic elliptical vibration boring elucidates this phenomenon. As speed increases, the cutting speed of the tool progressively rises in the cutting direction, approaching the critical speed. This diminishes the occurrence of tool–workpiece separation and reduces its impact on cutting force. Consequently, it augments the energy input into the turning system, weakens the damping effect of ultrasonic vibration on tool bar chatter, and compromises the stability of the turning system. The stiffness of the machining system decreases with escalating rotational speed, leading to diminished surface accuracy and an increase in the roundness value of deep holes.

At higher speeds, although the roundness in two-dimensional ultrasonic vibration turning also experiences an increase, it remains lower than that achieved in conventional turning. As demonstrated in Figure 18, employing the same cutting parameters, two-dimensional ultrasonic vibration turning can achieve a maximum reduction of 54.4% in the roundness value of the machined deep hole surface, with an average reduction of 50.6%. Thus, selecting an appropriate spindle speed during actual machining processes can not only enhance machining efficiency but also ensure machining precision.

3. The Influence of Cutting Depth on Roundness

**Figure 19:**
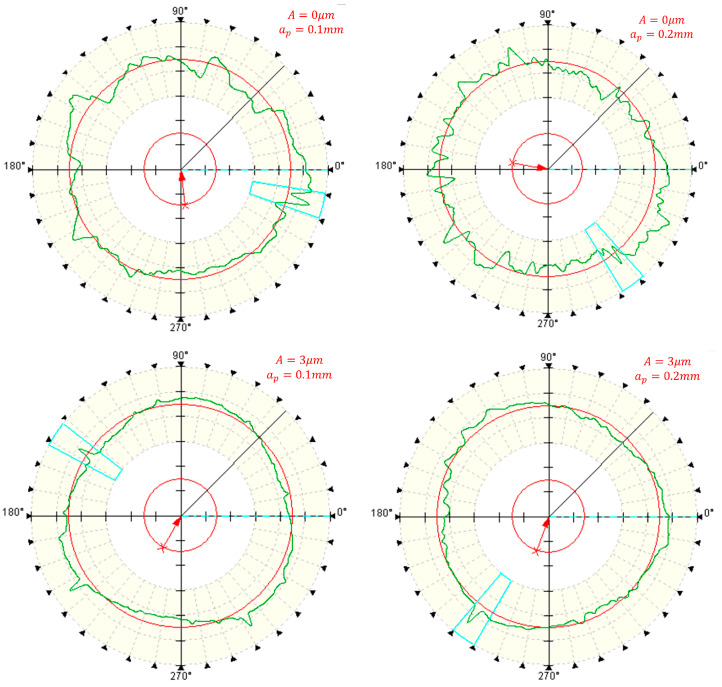
Measurement of roundness at different cutting depths.

**Figure 20:**
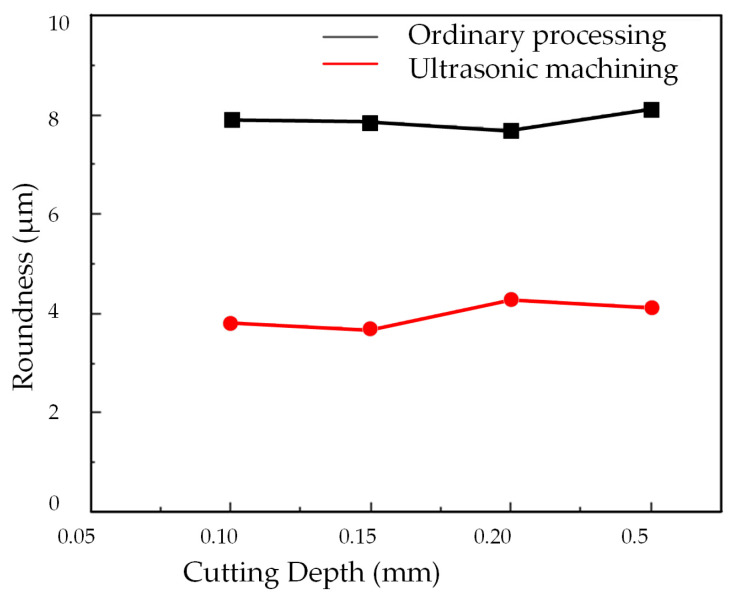
Changing the cutting depth.

Figure 19 presents the roundness error curve of deep holes following processing with varying cutting depths, while Figure 20 illustrates the impact of cutting depth on the surface roundness of the deep holes within the workpiece.

Figure 20 reveals that, in both conventional machining and two-dimensional ultrasonic vibration machining approaches, there is no substantial alteration in the roundness of the deep hole surface with increasing cutting depth. This suggests that the influence of cutting depth on the roundness of turned deep holes is not substantial.

However, it is worth noting that the roundness value of deep holes achieved through two-dimensional ultrasonic vibration turning is significantly smaller than that achieved through conventional turning. This aligns with the theoretical analysis findings of ultrasonic vibration cutting deep holes, underscoring the significant enhancement of machining system stiffness and surface accuracy facilitated by ultrasonic vibration. Consequently, in practical production scenarios, increasing the cutting depth can be considered to enhance machining efficiency.

4. The Effect of Ultrasonic Amplitude on Roundness

**Figure 21:**
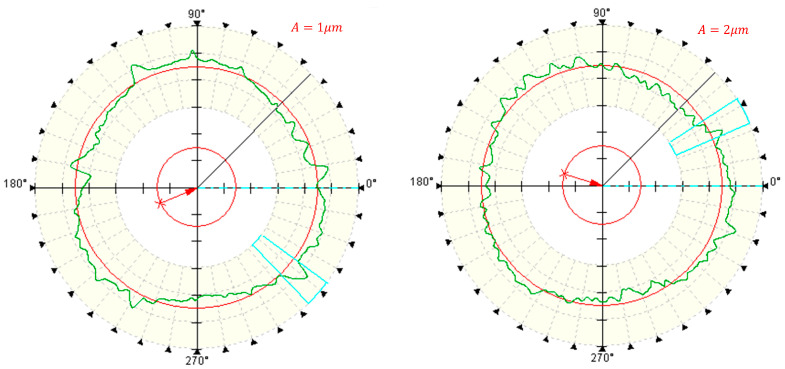

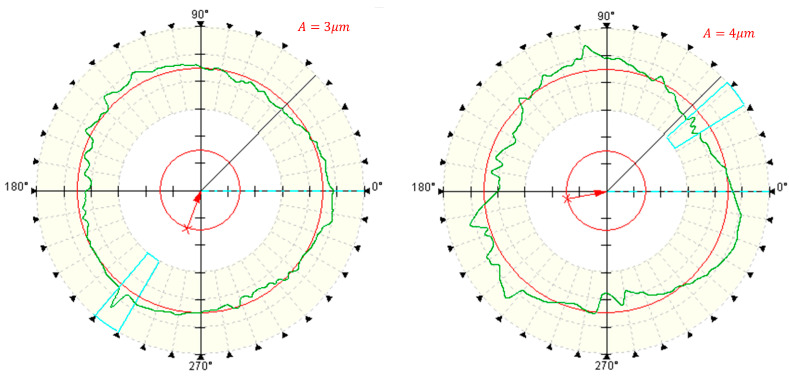
Measurement of roundness under different amplitudes.

**Figure 22:**
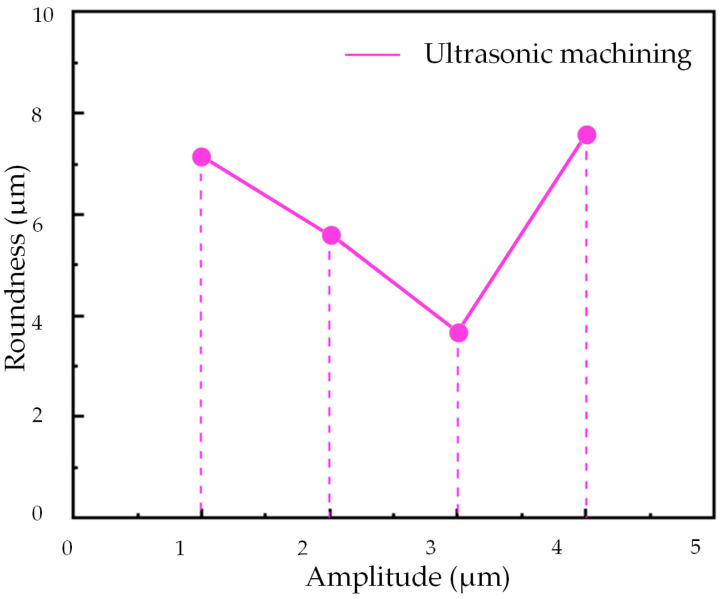
Different amplitudes.

Figure 21 and Figure 22 illustrate the impact of ultrasonic amplitude on the roundness of deep hole surfaces, similar to other machining parameters.

The roundness exhibits a pattern of initially decreasing and subsequently increasing as the ultrasonic amplitude increases. It reaches its minimum at an ultrasonic amplitude of 3 μm. As explained in the analysis of cutting edge motion trajectories in Section 2, it becomes evident that two-dimensional ultrasonic vibration turning demonstrates the phenomenon of rotary cutting, which becomes more pronounced with increasing amplitude and yields a more favorable effect in reducing cutting force. However, larger amplitudes can generate larger impacts, increase cutting force, increase energy intake of the turning system, and lead to a decrease in the stability of the machining system. Therefore, in actual processing and production, an appropriate ultrasonic amplitude should be selected.

### 4.2. Analysis of Roundness Orthogonal Test Results

**Figure 23:**
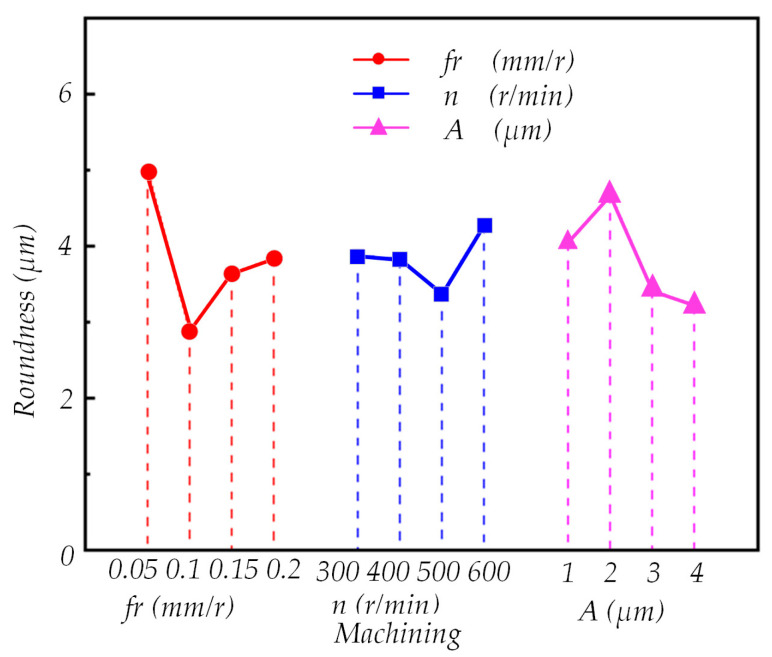
Effect of Different Processing Parameters on Roundness.

To investigate the impact of various processing parameters and acoustic factors on the roundness of the machined surface, range analysis and analysis of variance were conducted using the data presented in Table 6. The results are summarized in Table 7 and Table 8. Based on the findings from the range analysis, Figure 23 was generated, revealing that the optimal processing parameters are as follows: *f_r_* = 0.1 mm/r, *n* = 600 r/min, *A* = 4 μm, and *a_p_* = 0.1 mm.

As evident in Table 8, it is apparent that the mean square deviation of the cutting depth factor is smaller than the mean square deviation of the error, measuring 0.644819 compared with 0.801856. This suggests that the impact of the cutting depth factor on the roundness of the machined surface is relatively minor and should be categorized as an error. Therefore, the sum of the squared deviations, mean square deviation, and degrees of freedom of the error all need to be recalculated. The total squared error after recalculation is 1.446675, with a degree of freedom of 6. The mean square error after recalculation is 0.2411125. According to the table, $F_{0.05}\left(3,6\right)=4.76,\;F_{0.01}\left(3,6\right)=9.78$. By looking up the table, it can be seen that $F_{f_{r}}=1.182062>4.76,\;F_{A}=6.483327>4.76$.

Hence, at a significance level of α = 0.05, the impact on the roundness of the machined surface can be ranked as follows: feed rate > amplitude > speed > cutting depth. Both feed rate and ultrasonic amplitude exert a substantial influence on the roundness of the deep hole surface, accounting for 56.69% and 32.83%, respectively. The effect of rotational speed on roundness is 10.49%, but it is not deemed significant. Conversely, the influence of cutting depth on roundness is considered insignificant and relatively negligible, warranting minimal attention.

## 5. Conclusions

This research delves into the realm of two-dimensional ultrasonic elliptical vibration deep hole boring applied to 7075 aluminum alloy, with a particular focus on the analysis and investigation of the surface roundness achieved in the machining process. The primary conclusions drawn from this research are outlined below:

1. This approach begins with the design of a two-dimensional ultrasonic vibration boring tool featuring a single-excitation asymmetric structure. Subsequently, a model describing the motion trajectory of the cutting edge in two-dimensional ultrasonic elliptical vibration deep hole boring is developed, and a simulation analysis is conducted to explore the cutting edge’s behavior under various machining parameters. Through theoretical analysis and simulation analysis, the correctness and effectiveness of a two-dimensional ultrasonic vibration boring device with single-excitation asymmetric structure are proved.
2. The simulation results show that when the amplitude is A=4μ, the rotary cutting phenomenon is obvious, and the group SR&lt is satisfied. Under the condition of 1, the higher speed can improve the machining efficiency and ensure the two-dimensional ultrasonic vibration boring effect. The cutting depth has no obvious influence on the tool trajectory, and the feed rate only produces displacement change in the X direction.
3. An experimental platform for two-dimensional ultrasonic elliptical vibration deep hole boring was meticulously constructed. Both single-factor and orthogonal experimental plans were meticulously devised, and the ensuing experimental outcomes were meticulously measured and comprehensively analyzed.
4. The experimental results found that, firstly, as the feed rate escalates, the roundness of the deep hole surface experiences an increase. Secondly, an increase in spindle speed corresponds to an augmentation in the roundness of the deep hole surface. Notably, when the speed remains below 400 r/min, alterations in speed do not lead to significant changes in roundness. However, when the speed surpasses 400 r/min, there is a noticeable elevation in the roundness value of the deep hole surface. Thirdly, as the ultrasonic amplitude grows, the roundness of the deep holes exhibits an initial decrease followed by an increase. This suggests that larger amplitudes tend to decrease the roundness of deep holes. Lastly, the impact of cutting depth on the roundness of the deep hole surface is relatively insignificant. As the cutting depth continues to rise, there is a marginal increase in the roundness of the deep hole surface.
5. A comparative analysis was conducted between the results of ultrasonic boring and conventional boring experiments. The analysis revealed that the first processing method enhances the quality of deep hole machining, with a maximum roundness reduction of 54.1% and an average reduction of 50.4%. This underscores the significant impact of two-dimensional ultrasonic vibration boring on reducing the roundness value of deep hole machining. Based on the comprehensive orthogonal experimental findings, the optimal combination of processing parameters is as follows:
  fr=0.1 mm/r, n=600 r/min, A=4 μ m, and ap=0.1 mm

## Figures and Tables

**Table 1:** Motion trajectory simulation parameters.

Group Number	Spindle Speed *n* (r/min)	Cutting Depth *a_p_* (mm)	Feed Speed *v_f_* (mm/r)	Ultrasonic Amplitude *A* (μm)
1	200	0.15	0.1	3
	400			
	600			
	800			
2	600	0.1	0.1	3
		0.15		
		0.2		
		0.25		
3	600	0.15	0.05	3
			0.1	
			0.15	
			0.2	
4	600	0.15	0.1	1
				2
				3
				4

**Table 2:** Test Equipment and Conditions.

Equipment and Material Names	Parameter
CNC lathe	CK6140
Ultrasonic generator	Resonant frequency 20 kHz
Laser displacement sensor	LK-G10

**Table 3:** Tool parameters.

Material	Anterior Angle/(°)	Relief Angle/(°)	Nose Radius/mm	Nose Angle/(°)	Lead Angle/(°)	Blade Inclination Angle/(°)
hard metal	0	7	0.4	55	62.5	0

**Table 4:** L_16_ (4^4^) Orthogonal test table.

Level	*f_r_* (mm/r)	*n* (r/min)	*a_p_* (mm)	A(μm)
1	0.05	200	0.1	1
2	0.1	400	0.15	2
3	0.15	600	0.2	3
4	0.2	800	0.25	4

**Table 5:** Single-factor test table.

NO.	*f_r_* (mm/r)	*n* (r/min)	*a_p_* (mm)	A (μm)
E-1	0.05	400	0.15	3
E-2	0.1	400	0.15	3
E-3	0.15	400	0.15	3
E-4	0.2	400	0.15	3
F-1	0.1	200	0.15	3
F-2	0.1	400	0.15	3
F-3	0.1	600	0.15	3
F-4	0.1	800	0.15	3
G-1	0.1	400	0.1	3
G-2	0.1	400	0.15	3
G-3	0.1	400	0.2	3
G-4	0.1	400	0.25	3
H-1	0.1	400	0.15	1
H-2	0.1	400	0.15	2
H-3	0.1	400	0.15	3
H-4	0.1	400	0.15	4

**Table 6:** $L_{16}\left(4^{4}\right)$ Orthogonal test results.

Test Number	*f_r_* (mm/r)	*n* (r/min)	A (μm)	*a_p_* (mm)	RON (μm)
A-1	0.05	200	1	0.1	4.705
A-2	0.05	400	2	0.15	6.415
A-3	0.05	600	3	0.2	3.88
A-4	0.05	800	4	0.25	4.905
B-1	0.1	200	2	0.2	3.675
B-2	0.1	400	1	0.25	3.06
B-3	0.1	600	3	0.1	1.91
B-4	0.1	800	4	0.15	2.9
C-1	0.15	200	3	0.25	3.58
C-2	0.15	400	4	0.2	2.535
C-3	0.15	600	1	0.15	3.765
C-4	0.15	800	2	0.1	4.67
D-1	0.2	200	4	0.15	3.51
D-2	0.2	400	3	0.1	3.275
D-3	0.2	600	2	0.25	3.905
D-4	0.2	800	1	0.2	4.62

**Table 7:** RON Range Analysis.

Level	Feed Rate	Speed	Amplitude	Cutting Depth
K1	4.9763	3.8675	4.0375	3.64
K2	2.8863	3.8213	4.6663	4.1475
K3	3.6375	3.365	3.4088	3.6775
K4	3.8275	4.2738	3.215	3.8625
Range R	2.09	0.9088	1.4513	0.5075

**Table 8:** RON analysis of variance.

Factor	Sum of Squares of Deviations	Freedom	Mean Square Error	F Compare	Contribution Rate	Significance
*f_r_*	8.966406	3	2.988802	11.182062	56.69%	0.004892
*n*	1.658431	3	0.552810	2.068240	10.49%	0.226598
*A*	5.192131	3	1.730710	6.475140	32.83%	0.068421
*a_p_*	0.644819	3	0.214940	0.804158		0.656485
Error column	0.801856	3	0.267285			

According to the table: F_0.05_(3,6) = 4.76; F_0.01_(3,6) = 9.78.

## Data Availability

Data are contained within the article.
